# Supplementary material for: Species abundances surpass richness effects in the biodiversity-ecosystem function relationship across marine fishes
Source: Nat Commun. 2025 Aug 21;16:7789. doi: 10.1038/s41467-025-63210-x (PMC12371026; doi:10.1038/s41467-025-63210-x)
Supplement: Supplementary file 1 — Supplementary information [file 41467_2025_63210_MOESM1_ESM.pdf]

***Supplementary information for*:**

Species abundances surpass richness effects in the biodiversity-ecosystem function relationship across marine fishes

Nature Communications

Helen F. Yan[1,2]*, Renato A. Morais[3], David R. Bellwood[1]

[1]*Research Hub for Coral Reef Ecosystem Functions, College of Science and Engineering, James Cook University, Townsville, QLD 4811, Australia*
[2]*Thriving Oceans Research Hub, School of Geosciences, University of Sydney, Camperdown, NSW 2006, Australia*
[3]*Paris Sciences et Lettres Université, École Pratique des Hautes Études, EPHE-UPVD-CNRS, USR 3278 CRIOBE, Perpignan, 66860, France*

*Corresponding author: Helen F. Yan, Thriving Oceans Research Hub, University of Sydney, Camperdown, NSW 2006, Australia.
Email: helen.yan@sydney.edu.au

Supplementary figures

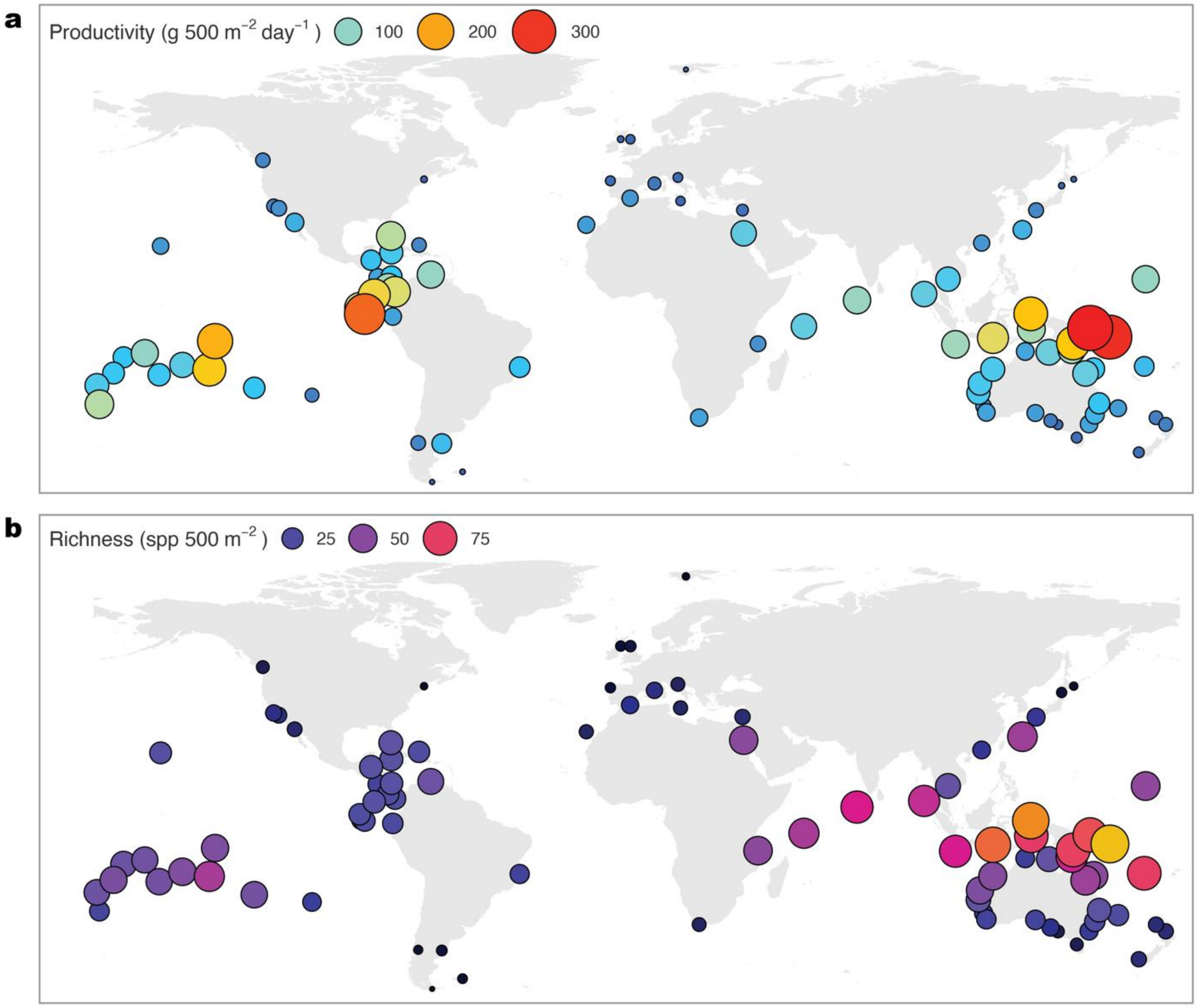

FIGURE S1. Global patterns of marine fish productivity and richness. a Fish biomass production (g 500 m^{-2} day^{-1}) of all surveyed marine teleost fishes whereby the size and colour of the points denote the magnitude of productivity. **b** Global distribution of species richness (number of species per 500 m^{-2}); the points are sized and coloured based on the number of species per survey. Each point is the median value per ecoregion. Baseline map downloaded from https://geonode.wfp.org/layers/geonode%3Awld_bnd_adm0_wfp. Source data are provided as a Source Data file.

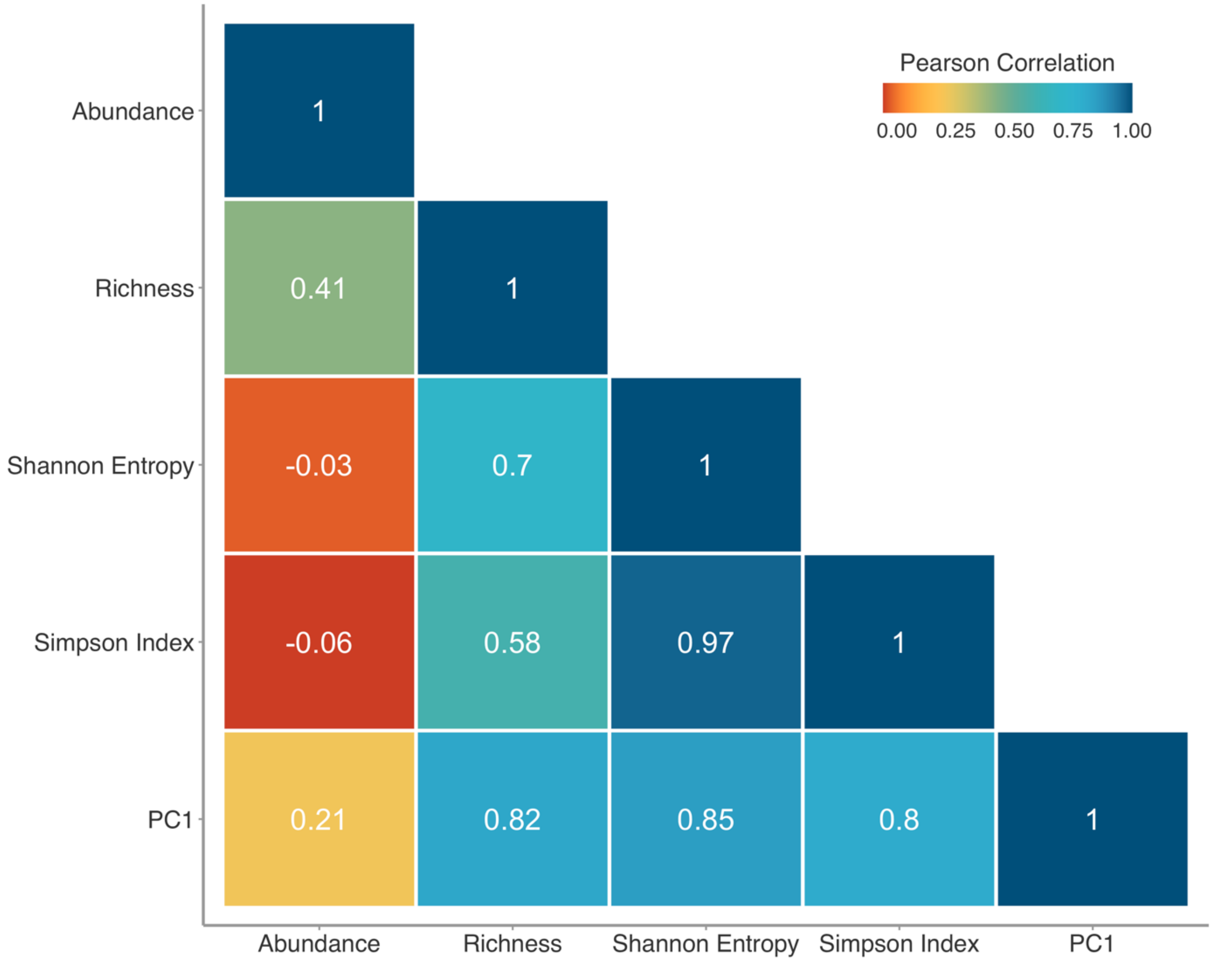

FIGURE S2. Correlation heatmap of different Hill diversity metrics with principal component axis 1 (PC1, as seen in Fig. 1a). Tiles in red/orange denote negative correlations, while cooler colours denote positive correlations. Source data are provided as a Source Data file.

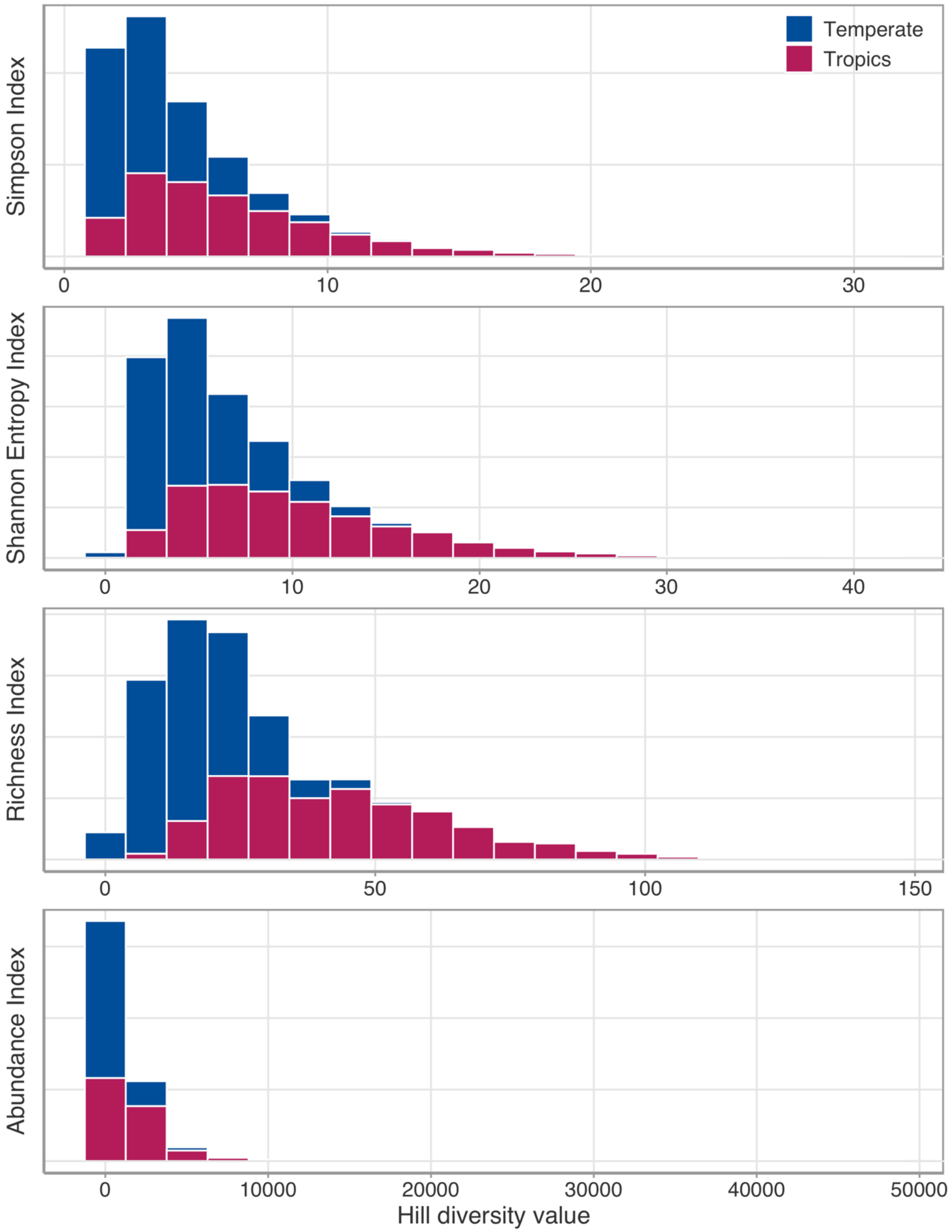

FIGURE S3. Hill diversity gradients across geographic regions. Simpson Index (ℓ = -1), Shannon Entropy Index (ℓ = 0), richness index (ℓ = 1), and abundance index (ℓ = 10) gradients across temperate (blue) and tropical (red) locations. All diversity indices were logged prior to model fitting. Source data are provided as a Source Data file.

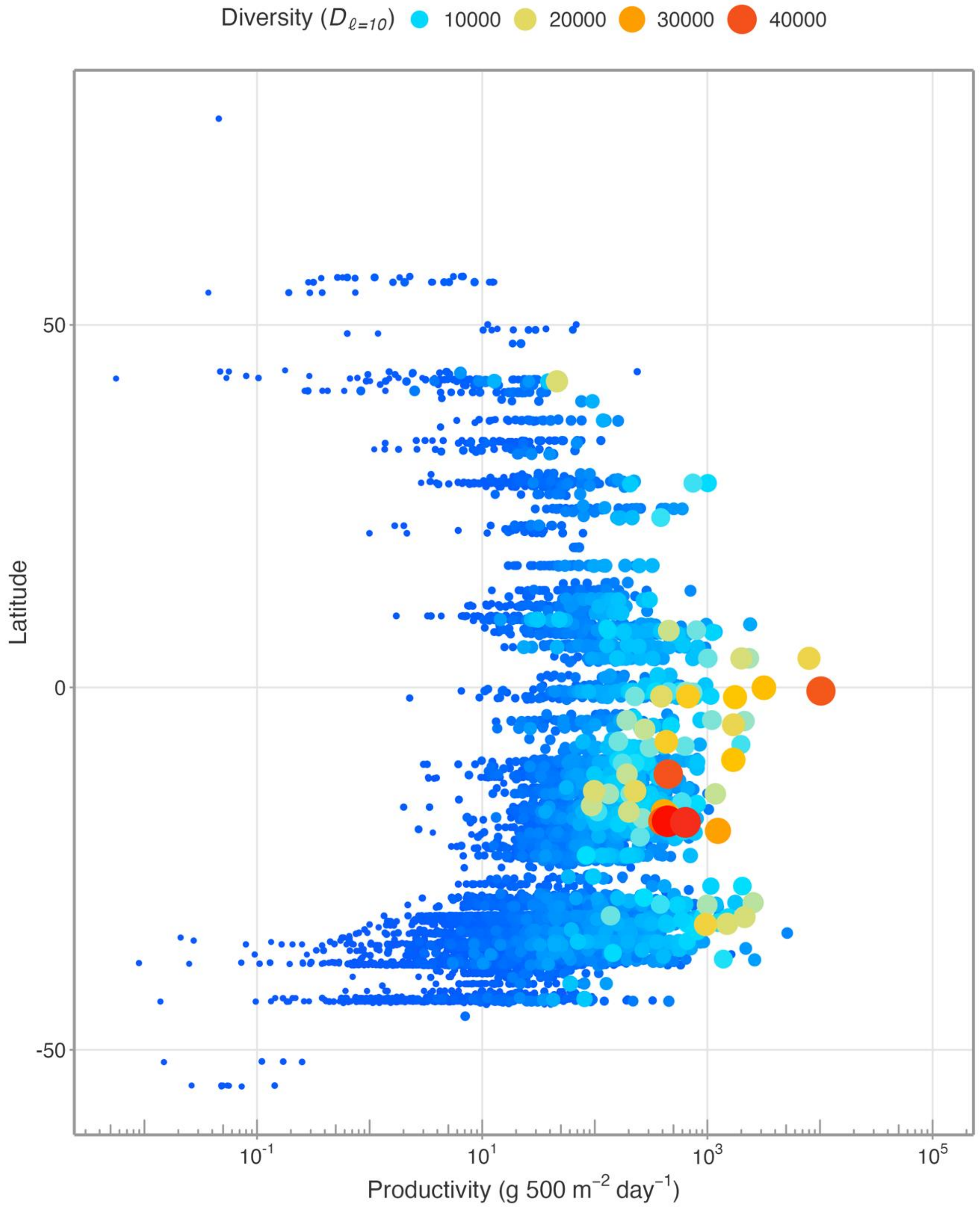

FIGURE S4. Latitudinal patterns of reef fish productivity. Patterns of fish productivity (g 500 m^{-2} day^{-1}) across latitudinal gradients. Points are sized and coloured based on their respective Hill diversity values emphasising abundance effects. Note the x axis is on the $\log_{10}$ scale. Source data are provided as a Source Data file.

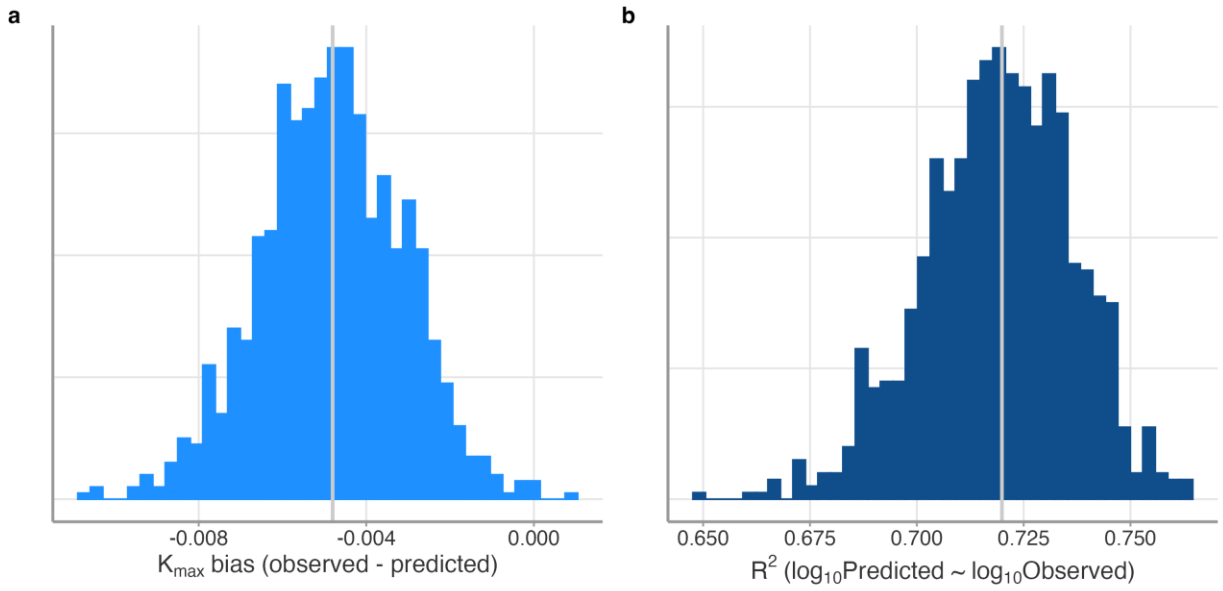

FIGURE S5. Extreme Gradient Boosting model diagnostics. a Prediction biases of K_{max} values across 1,000 bootstrapped iterations. Bias was calculated by subtracting the predicted values from the observed values. **b** The predicted R^2 value by fitting the $\log_{10}$Predicted values from the $\log_{10}$Observed values. Source data are provided as a Source Data file.

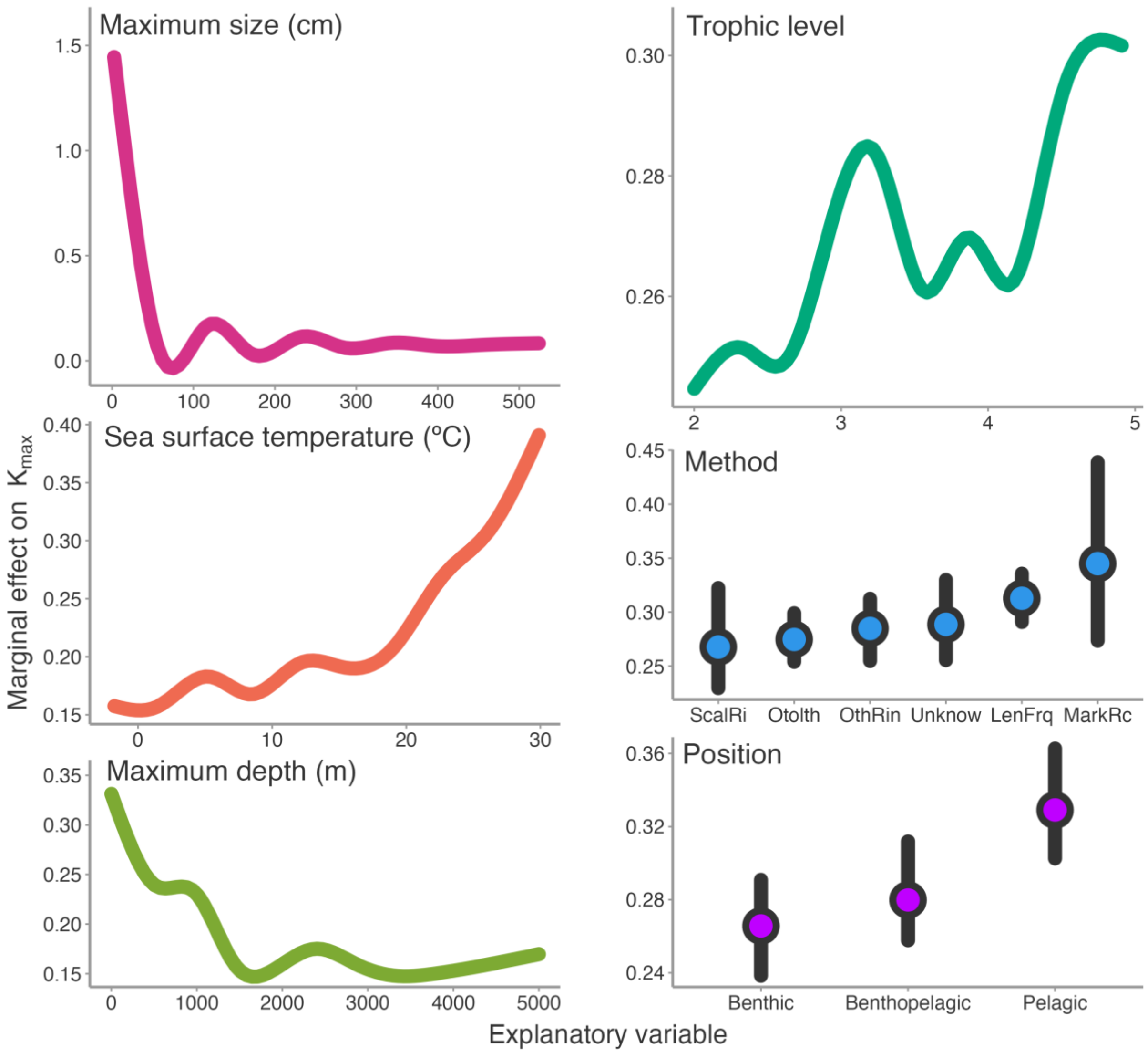

FIGURE S6. Predictors of fish growth. Partial dependence plots showing the effects of maximum body size (cm), sea surface temperature (ºC), maximum depth (m), trophic level, aging method, and position in the water column on K_{max} from 1,000 bootstrapped Extreme Gradient Boosting models. ScalRi = scale rings, Otolth = otoliths, OthRin = other rings, Unknow = unknown, LenFrq = length frequency, MarkRc = mark recapture. Source data are provided as a Source Data file.

Supplementary tables:

TABLE S1. Principal Component Analysis (PCA) results showing the loadings of the inverted Simpson Index, exponentiated Shannon entropy, species richness, and abundance effects on all four principal components. The proportion of variation explained by each axis is denoted in the parentheses. Source data are provided as a Source Data file.

**Dimension**	**PC1** (68.4%)	**PC2** (28.9%)	**PC3** (2.5%)	**PC4** (0.3%)
Simpson Index	0.52	-0.45	-0.45	-0.57
Shannon Index	0.56	-0.36	0.05	0.75
Species richness	0.55	0.32	0.71	-0.3
Abundance	0.34	0.76	-0.54	0.14

TABLE S2. Estimated fixed effects from model outputs of the regional BEF testing different Hill diversity metrics (D_ℓ) and per-capita biomass production generalised linear mixed-effects models (region = temperate or tropical). Coefficient estimates are shown as the median of the posterior distribution; lower and upper refer to the bounds of the 90% credible intervals (CI). Leave-one-out information criterion (LOOIC ± standard error) is shown in parentheses for competing BEF models using different Hill diversity metrics. ESS = bulk effective sample size.

Coefficients	Estimate	Lower CI	Upper CI	ESS
BEF with abundance index (89533.3 ± 351.7)				
Intercept	3.99	3.56	4.39	2295
Log ($D_{\ell=10}$)	1.08	1.06	1.10	4685
Tropics	-0.43	-0.98	-0.15	2286
Tropics * log ($D_{\ell=10}$)	0.074	0.033	0.11	4616
Sea surface temperature	0.21	0.17	0.25	2508
Visibility	0.060	0.039	0.080	3881
Depth	0.025	0.0093	0.041	4535
BEF with species richness (93866.9 ± 356.2)				
Intercept	4.46	3.95	4.97	2002
Log ($D_{\ell=1}$)	1.08	1.04	1.12	4009
Tropics	-0.81	-1.42	-0.13	2268
Tropics * log ($D_{\ell=1}$)	-0.11	-0.18	-0.045	3193
Sea surface temperature	0.25	0.19	0.30	2176
Visibility	0.042	0.016	0.071	3782
Depth	0.077	0.058	0.098	4406
BEF with exponentiated Shannon entropy (95849.4 ± 328.7)				
Intercept	4.06	3.75	4.37	2442
Log ($D_{\ell=0}$)	-0.088	-0.12	-0.055	4032
Tropics	-0.66	-1.09	-0.21	2698
Tropics * log ($D_{\ell=0}$)	-0.040	-0.087	0.0093	3540
Sea surface temperature	1.04	0.99	1.10	1805
Visibility	0.14	0.11	0.17	3318
Depth	0.082	0.058	0.11	3932
BEF with inverse Simpson Index (95852.8 ± 328.9)				
Intercept	4.05	3.73	4.36	2667
Log ($D_{\ell=-1}$)	-0.11	-0.13	-0.075	5114
Tropics	-0.66	-1.08	-0.22	3033
Tropics * log ($D_{\ell=-1}$)	-0.0088	-0.050	0.038	4581
Sea surface temperature	1.04	0.98	1.10	2210
Visibility	0.14	0.11	0.17	4433
Depth	0.084	0.061	0.11	4759

Per-capita model with abundance index				
Intercept	-3.16	-3.47	-2.88	2829
Log ($D_{\ell = 10}$)	-0.33	-0.35	-0.31	4763
Tropics	-0.18	-0.57	0.24	2825
Tropics * log ($D_{\ell = 10}$)	-0.080	-0.12	-0.041	4657
Sea surface temperature	0.23	0.18	0.27	2472
Visibility	0.063	0.042	0.083	4602
Depth	0.022	0.0062	0.037	4954
Abundance model with abundance index				
Intercept	7.15	6.87	7.43	2828
Log ($D_{\ell = 10}$)	1.42	1.41	1.43	5597
Tropics	-0.24	-0.63	0.15	3052
Tropics * log ($D_{\ell = 10}$)	0.14	0.13	0.16	3052
Sea surface temperature	-0.037	-0.055	-0.019	2147
Visibility	-0.0060	-0.015	0.0027	3900
Depth	-0.0018	-0.0083	0.0043	5583
BEF with abundance index and Boltzmann temperature				
Intercept	3.99	3.55	4.41	2501
Log ($D_{\ell = 10}$)	1.08	1.06	1.10	5076
Tropics	-0.44	-0.98	0.17	2381
Tropics * log ($D_{\ell = 10}$)	0.075	0.035	0.12	4546
Boltzmann temperature	-0.22	-0.26	-0.17	2574
Visibility	0.059	0.038	0.081	4538
Depth	0.025	0.0082	0.041	4810

TABLE S3. Variable importance (%) of explanatory variables used to predict K_{max} across fishes shown as the median, minimum, and maximum across 1,000 bootstrapped models. Source data are provided as a Source Data file.

Variable	Median	Minimum	Maximum
Maximum body size	43.1	38.8	47.5
Sea surface temperature	21.1	18.5	23.8
Maximum depth	14.6	12.5	17.5
Trophic level	12.1	10.2	13.9
Aging method	5.3	4.1	6.8
Position in the water column	3.7	3.0	4.9
