# Supplementary material for: Species abundances surpass richness effects in the biodiversity-ecosystem function relationship across marine fishes
Source: Nat Commun. 2025 Aug 21;16:7789. doi: 10.1038/s41467-025-63210-x (PMC12371026; doi:10.1038/s41467-025-63210-x)
Supplement: Supplementary file 2 — Reporting Summary [file 41467_2025_63210_MOESM2_ESM.pdf]

nature portfolio

Corresponding author(s): Helen F. Yan
Last updated by author(s): 11 May 2025

Reporting Summary

Nature Portfolio wishes to improve the reproducibility of the work that we publish. This form provides structure for consistency and transparency in reporting. For further information on Nature Portfolio policies, see our Editorial Policies and the Editorial Policy Checklist.

Statistics

For all statistical analyses, confirm that the following items are present in the figure legend, table legend, main text, or Methods section.

n/a	Confirmed	
☐	☒	The exact sample size (n) for each experimental group/condition, given as a discrete number and unit of measurement
☐	☒	A statement on whether measurements were taken from distinct samples or whether the same sample was measured repeatedly
☐	☒	The statistical test(s) used AND whether they are one- or two-sided<br>*Only common tests should be described solely by name; describe more complex techniques in the Methods section.*
☐	☒	A description of all covariates tested
☐	☒	A description of any assumptions or corrections, such as tests of normality and adjustment for multiple comparisons
☐	☒	A full description of the statistical parameters including central tendency (e.g. means) or other basic estimates (e.g. regression coefficient) AND variation (e.g. standard deviation) or associated estimates of uncertainty (e.g. confidence intervals)
☒	☐	For null hypothesis testing, the test statistic (e.g. F, t, r) with confidence intervals, effect sizes, degrees of freedom and P value noted<br>*Give P values as exact values whenever suitable.*
☐	☒	For Bayesian analysis, information on the choice of priors and Markov chain Monte Carlo settings
☐	☒	For hierarchical and complex designs, identification of the appropriate level for tests and full reporting of outcomes
☒	☐	Estimates of effect sizes (e.g. Cohen's d, Pearson's r), indicating how they were calculated

Our web collection on statistics for biologists contains articles on many of the points above.

Software and code

Policy information about availability of computer code

Data collection	No custom software or code was used for data collection.
Data analysis	All analyses were run on macOS 14.6.1 in R v 4.1.0 using the following R packages: brms (2.16.4), DHARMa (0.4.5), XGBoost (1.4.1.1), performance (0.12.0), rstan (2.32.6), truncnorm (1.0-9), doSNOW (1.0.20), snow (0.4-4), foreach (1.5.2), MeanRarity (0.0.1.0005), raster (3.6-26), sp (2.1-4), sf (1.0-16), patchwork (1.2.0), fishualize (0.2.3), Cairo (1.6-2), tidyverse (2.0.0)

For manuscripts utilizing custom algorithms or software that are central to the research but not yet described in published literature, software must be made available to editors and reviewers. We strongly encourage code deposition in a community repository (e.g. GitHub). See the Nature Portfolio guidelines for submitting code & software for further information.

Data

Policy information about availability of data

All manuscripts must include a data availability statement. This statement should provide the following information, where applicable:

- Accession codes, unique identifiers, or web links for publicly available datasets
- A description of any restrictions on data availability
- For clinical datasets or third party data, please ensure that the statement adheres to our policy

The data and code in this study are available at Figshare (https://doi.org/10.6084/m9.figshare.26156344). There are no restrictions on data availability. The publicly available survey data used in the main analyses was downloaded from The Reef Life Survey Program (https://

reeflifesurvey.com/), FishBase (http://www.fishbase.org), and the data repositories of refs. 38 (https://zenodo.org/records/7797270) and 69 (https://doi.org/10.4225/28/5ae8f3cc790f9).

Research involving human participants, their data, or biological material

Policy information about studies with human participants or human data. See also policy information about sex, gender (identity/presentation), and sexual orientation and race, ethnicity and racism.

Reporting on sex and gender	N/A
Reporting on race, ethnicity, or other socially relevant groupings	N/A
Population characteristics	N/A
Recruitment	N/A
Ethics oversight	N/A

Note that full information on the approval of the study protocol must also be provided in the manuscript.

Field-specific reporting

Please select the one below that is the best fit for your research. If you are not sure, read the appropriate sections before making your selection.

☐ Life sciences ☐ Behavioural & social sciences ☒ Ecological, evolutionary & environmental sciences

For a reference copy of the document with all sections, see nature.com/documents/nr-reporting-summary-flat.pdf

Ecological, evolutionary & environmental sciences study design

All studies must disclose on these points even when the disclosure is negative.

Study description	We analysed the biodiversity-ecosystem function relationship (BEF) across marine fishes globally. We used an extreme gradient boosting algorithm with estimates of somatic growth mined from the peer-reviewed literature to generate predictive estimates of somatic growth. We then calculated biomass production as the biomass expected via somatic growth of all individuals surveyed from the Reef Life Survey program. We then assessed the effects of biodiversity and species' abundances on the biomass production of fishes spanning polar to tropical locations.
Research sample	The research sample used in this study comprises teleost fishes for which there were previously published estimates of empirical growth and those that were observed through the Reef Life Survey program. The rationale for our sample choice was that we were interested in exploring the biodiversity-ecosystem function relationship across as many marine teleost fishes as possible. Most of the growth data is publicly available through FishBase, but required some corrections from the peer-reviewed literature (see Data collection).
Sampling strategy	No statistical methods were used to predetermine sample size. All sample sizes were limited by the availability of published growth parameters and species that were visually detected along transects in the Reef Life Survey program.
Data collection	All data were collected from the publicly available online repositories including FishBase (fishbase.org), the Reef Life Survey program (reeflifesurvey.com), and published literature.
Timing and spatial scale	The spatial scale of this study is global. Because all data used were publicly available, there were no time constraints for data collection.
Data exclusions	We excluded all transects that were only composed of a single block.
Reproducibility	There were no experiments involved in our study; therefore, our analyses do not contain replicates. We provide all relevant data (and data sources) and code to reproduce the results and figures from this study.
Randomization	We did not randomize any aspect of the study because we did not conduct any experiments. All organisms included were chosen based on their habitat and taxonomic affinities: marine teleost fishes.
Blinding	No experiment was conducted, therefore blinding was not relevant.

Did the study involve field work? ☐ Yes ☒ No

Reporting for specific materials, systems and methods

We require information from authors about some types of materials, experimental systems and methods used in many studies. Here, indicate whether each material, system or method listed is relevant to your study. If you are not sure if a list item applies to your research, read the appropriate section before selecting a response.

Materials & experimental systems

n/a	Involved in the study
☒	☐ Antibodies
☒	☐ Eukaryotic cell lines
☒	☐ Palaeontology and archaeology
☒	☐ Animals and other organisms
☒	☐ Clinical data
☒	☐ Dual use research of concern
☒	☐ Plants

Methods

n/a	Involved in the study
☒	☐ ChIP-seq
☒	☐ Flow cytometry
☒	☐ MRI-based neuroimaging

Plants

Seed stocks	N/A
Novel plant genotypes	N/A
Authentication	N/A
